# ATF5 regulates tubulointerstitial injury in diabetic kidney disease via mitochondrial unfolded protein response

**DOI:** 10.1186/s10020-023-00651-4

**Published:** 2023-04-24

**Authors:** Yifei Liu, Lei Zhang, Shumin Zhang, Jialu Liu, Xiaohui Li, Kexin Yang, Danyi Yang, Yu Liu, Lin Sun, Fuyou Liu, Li Xiao

**Affiliations:** Department of Nephrology, The Second Xiangya Hospital, Central South University, Changsha, 410011 Hunan China

**Keywords:** Diabetic kidney disease, Tubular cell, ATF5, UPRmt, Apoptosis, Oxidative stress

## Abstract

**Background:**

Mitochondrial quality control (MQC) plays a critical role in the progression of tubulointerstitial injury in diabetic kidney disease (DKD). The mitochondrial unfolded protein response (UPRmt), which is an important MQC process, is activated to maintain mitochondrial protein homeostasis in response to mitochondrial stress. Activating transcription factor 5 (ATF5) is critical in the mammalian UPRmt via mitochondria-nuclear translocation. However, the role of ATF5 and UPRmt in tubular injury under DKD conditions is unknown.

**Methods:**

ATF5 and UPRmt-related proteins including heat shock protein 60 (HSP60) and Lon peptidase 1 (LONP1), in DKD patients and db/db mice were examined by immunohistochemistry (IHC) and western blot analysis. Eight-week-old db/db mice were injected with ATF5-shRNA lentiviruses via the tail vein, and a negative lentivirus was used as a control. The mice were euthanized at 12 weeks, and dihydroethidium (DHE) and TdT-mediated dUTP nick end labeling (TUNEL) assays were performed to evaluate reactive oxygen species (ROS) production and apoptosis in kidney sections, respectively. In vitro, ATF5-siRNA, ATF5 overexpression plasmids or HSP60-siRNA were transfected into HK-2 cells to evaluate the effect of ATF5 and HSP60 on tubular injury under ambient hyperglycemic conditions. Mitochondrial superoxide (MitoSOX) staining was used to gauge mitochondrial oxidative stress levels, and the early stage of cell apoptosis was examined by Annexin V-FITC kits.

**Results:**

Increased ATF5, HSP60 and LONP1 expression was observed in the kidney tissue of DKD patients and db/db mice and was tightly correlated with tubular damage. The inhibition of HSP60 and LONP1, improvements in serum creatinine, tubulointerstitial fibrosis and apoptosis were observed in db/db mice treated with lentiviruses carrying ATF5 shRNA. In vitro, the expression of ATF5 was increased in HK-2 cells exposed to high glucose (HG) in a time-dependent manner, which was accompanied by the overexpression of HSP60, fibronectin (FN) and cleaved-caspase3 (C-CAS3). ATF5-siRNA transfection inhibited the expression of HSP60 and LONP1, which was accompanied by reduced oxidative stress and apoptosis in HK-2 cells exposed to sustained exogenous high glucose. ATF5 overexpression exacerbated these impairments. HSP60-siRNA transfection blocked the effect of ATF5 on HK-2 cells exposed to continuous HG treatment. Interestingly, ATF5 inhibition exacerbated mitochondrial ROS levels and apoptosis in HK-2 cells in the early period of HG intervention (6 h).

**Conclusions:**

ATF5 could exert a protective effect in a very early stage but promoted tubulointerstitial injury by regulating HSP60 and the UPRmt pathway under DKD conditions, providing a potential target for the prevention of DKD progression.

**Supplementary Information:**

The online version contains supplementary material available at 10.1186/s10020-023-00651-4.

## Background

Diabetic kidney disease (DKD) is one of the most common complications and the leading cause of end-stage renal disease (ESRD) worldwide, with an approximately 40% incidence (Zhang et al. 2016; Koye et al. 2018). Many studies have demonstrated that proximal tubular injury contributes to the progression of DKD and tightly correlates with a decline in renal function (Gilbert 2017; Tang et al. 2011; Zeni et al. 2017). Renal proximal tubular cells are rich in mitochondria, sensitive to metabolic disturbances and abnormal energy supply, and prone to developing mitochondrial dysfunction under hyperglycemic conditions (Lee et al. 2019), Clinical studies have shown relatively fewer mitochondria and lower ATP production in type 2 diabetes mellitus patients (T2DM) than in healthy controls (Nunnari and Suomalainen 2012; Morino et al. 2005). Changes in mitochondrial function result in insulin resistance and a continuous decline in renal function in DKD (Petersen et al. 2004; Aluksanasuwan et al. 2017a). Fragmented mitochondria in renal tubular cells were observed in a diabetic animal model and DKD patients (Blake and Trounce 2014; Ahmad et al. 2021). Oxidative stress in mitochondria blocks electron transport and disturbs tubular cell proteostasis and metabolism, subsequently triggering programmed cell death under DKD conditions (Petersen et al. 2004; Aluksanasuwan et al. 2017a; Wanagat and Hevener 2016). Thus, mitochondrial quality control (MQC) has attracted broad attention to maintain integral mitochondria and ameliorate tubular injury in DKD.

MQC ensures correct protein translation, folding, turnover and degradation in mitochondria (Voos et al. 2016). The regulation of MQC involves complex processes, such as chaperone modulation, protein sorting, ubiquitin‒proteasome degradation, fission/fusion dynamics, the mitochondrial unfolded protein response and mitophagy (Voos et al. 2016; Zhan et al. 2015). In our previous study and other studies, abnormal mitochondrial membrane potential, decreased ATP levels and increased ROS production, as well as mitochondrial fragmentation, were observed in proximal tubular cells treated with high glucose in vivo and in vitro, and these effects were closely correlated with tubular injury (Coughlan et al. 2016; Xiao et al. 2014; Sun et al. 2010; Czajka and Malik 2016; Huang et al. 2021). It was found that small GTPase, Rap1b and mitoQ could alleviate tubular cell apoptosis and tubulointerstitial fibrosis in DKD by improving mitochondrial function (Xiao et al. 2014, 2017). Moreover, abnormal mitophagy was observed and contributed to tubular cell apoptosis in DKD. Overall, failure to maintain proper MQC is the key to oxidative stress and tubular cell injury, which lead to the progression of DKD (Galvan et al. 2021).

The mitochondrial unfolded protein response (UPRmt) is a newly discovered process associated with MQC and various cell stresses (Lim et al. 2021). In response to mitochondrial stress, the degradation of denatured or misfolded proteins is blocked, and the transcription of nuclear gene-encoded proteins such as chaperones heat shock protein 60 (HSP60), heat shock protein 10 (HSP10) and proteases Lon peptidase 1 (LONP1) and caseinolytic protease (ClpP) is initiated to maintain mitochondrial proteostasis (Haynes et al. 2013). Recent studies have shown that the UPRmt is tightly correlated with aging, innate immunity, erythrocyte differentiation and the pathogenesis of cancer, infection and metabolic disorders (Martinus et al. 1996; Pellegrino and Haynes 2015; Shpilka and Haynes 2018; Fiorese and Haynes 2017). More recently, aberrant UPRmt was observed under diabetic conditions and was characterized by the downregulation of Hsp60, Hsp10, ClpP, and Lonp1 in C57BL/6N mice (Wardelmann et al. 2019). In addition, chronic hyperglycemia could impair the UPRmt and induce proteotoxicity in experimental diabetic neuropathy (Kalvala et al. 2020). However, the role of the UPRmt in tubular damage under diabetic conditions is not well known.

The mechanism of the UPRmt and its regulation is complex. It was verified that activating transcription factor associated with stress-1 (ATFS-1), a bZip transcription factor, could regulate the UPRmt in response to mitochondrial stress through the mitochondrial and nuclear targeting sequence in *C. elegans* (Nargund et al. 2012). Under physiological conditions, ATFS-1 enters mitochondria through the mitochondrial targeting sequence (MTS), which is cleaved and degraded by LONP1 in mitochondria. Under mitochondrial stress conditions, the mitochondrial import of ATFS-1 is decreased, and ATFS-1 accumulates in the cytoplasm, translocate into the nucleus through the nuclear localization sequence (NLS), and activates the transcription of UPRmt-related proteins, such as mitochondrial chaperones and proteases, to reestablish protein homeostasis and maintain normal mitochondrial function. It was suggested that UPRmt activation in mammalian cells also relies on the bZip transcription factor, and the mechanism might resemble that of *C. elegans* (Fiorese et al. 2016). However, the mammalian UPRmt regulatory mechanism is far more complex and remains unclear.

In 2016, activating transcription factor 5 (ATF5), an ortholog of ATFS-1, was shown to have a comparable function to ATFS-1 in the mammalian UPRmt (Fiorese et al. 2016). In response to mitochondrial stress, ATF5 translocates from the cytoplasm to the nucleus and induces transcription of the UPRmt gene cluster, thereby upregulating HSP60 and HSP10 levels and increasing ClpP expression to promote the degradation of misfolded proteins, indicating an activated UPRmt (Zhao et al. 2002). Based on ChIP analysis, ATF5 has been proven to be the downstream target of pancreatic duodenal homeobox-1 (Pdx1), which is an important pathogenic gene in diabetes. Moreover, knockdown of ATF5 rescued pancreatic β cells from stress-induced apoptosis (Juliana et al. 2017). However, whether ATF5 activates the UPRmt in mammalian DKD tubules to reduce tubular cell apoptosis needs to be elucidated.

In this study, we observed the role of ATF5-modulated UPRmt in tubular injury under diabetic conditions and investigated the underlying mechanisms by regulating HSP60; related content has been received in the World Congress of Nephrology 2022 (Liu et al. 2022).

## Materials and methods

### Animal study

Eight-week-old male db/db mice and age-matched db/m mice were purchased from the Aier Matt Experimental Animal Company (Suzhou, China) and randomly assigned into four groups (n = 6 in each group): control (db/m), db/db, db/db injected with negative control lentivirus (LV-NC), and db/db injected with the ATF5-shRNA lentivirus [LV-shRNA-ATF5, target sequence: tgACGGCTTCTCTGATTGGAT, constructed by GeneChem (Shanghai, China)], the transduction efficiency of the Bumpt cells (mouse renal tubular epithelial cells) transfected with LV-shRNA was showed in the Additional file 1: Fig. S1. The mice were maintained under a 12-h light–dark cycle in the Second Xiangya Hospital of Central South University and were given free access to tap water and commercial chow. The ATF5-shRNA lentivirus or negative control lentivirus was injected into eight-week-old db/db mice via the tail vein [the final lentiviral vector titers were determined to contain 3 × 10^8^ TU/mouse according to reported previously (Liu et al. 2016; Yang et al. 2020)]. Body weight was monitored weekly for four weeks. At 12 weeks of age, the mice were euthanized by an intraperitoneal injection of sodium pentobarbital (50 mg/kg body weight), and kidneys and blood samples were harvested. All animal procedures in this study were approved by the Animal Care and Use Committee of Second Xiangya Hospital of Central South University (Ethical approval number: 2020682).

### Blood and urine biochemistry

Blood glucose levels were evaluated weekly by a blood glucose monitor and test strips. Serum creatinine was measured using enzymatic methods (Hitachi 912 automated analyzer). Twenty-four-hour urine was obtained using metabolic cages once per week and was centrifuged at 3,000 rcf for 10 min at 4 °C to remove the sediment. Urine albumin concentrations were assessed by ELISA kits (Bethyl Laboratories). The urinary albumin‐creatinine ratio (UACR) was calculated as microalbumin divided by creatinine and is expressed in micrograms per milligrams.

### Morphological analysis and immunohistochemistry

Twenty patients were enrolled in this study. Human kidney biopsy sections were acquired from ten biopsy-proven type 2 DKD patients and ten patients with glomerular minor lesions who were recruited as controls (Gu et al. 2022). Among the 10 DKD patients, 3 were classified as stage II, 5 were classified as stage III and 2 were classified as stage IV according to the 2010 pathological classification of DKD (Tervaert et al. 2010). Patients who had taken immunosuppressive agents or adrenal cortical hormones were excluded. Our study was implemented with the approval of the Ethics Committee of Second Xiangya Hospital, Central South University in accordance with the Declaration of Helsinki (Ethics approval number: LYF2021179). Informed consent forms were obtained from all of the enrolled participants.

Kidney slices (4 μm) were stained with hematoxylin–eosin (HE), periodic acid-Schiff (PAS) and Masson trichrome staining (Masson). Interstitial fibrosis and tubular atrophy (IFTA) were evaluated based on cortical damage as follows: 0 = trivial interstitial fibrosis (< 5%); 1 = mild interstitial fibrosis (5–25%); 2 = moderate interstitial fibrosis (26–50%); and 3 = severe fibrosis (> 50%). Glomerular injury was evaluated by a semiquantitative scoring system, as previously described (Chen et al. 2019). After deparaffinize- tion and immersion in sodium citrate buffer to obtain antigen repair by being heated in a microwave, the kidney sections were blocked for 1 h. Then, the sections were incubated overnight at 4 °C with primary antibodies against ATF5 (Abcam, ab184923), HSP60 (Abcam, ab53109), LONP1 (Proteintech, 66043-1-Ig), fibronectin (Abcam, ab2413) and C-CAS3 (CST, 9664S) and were incubated with horseradish peroxidase-conjugated secondary antibodies for 1 h at room temperature. Following diaminobenzidine staining and hematoxylin restaining, the sections were dehydrated and cleared. Finally, we took images of the slides under a microscope and analyzed them with Image-Pro Plus (IPP) as previously reported (Qijiao et al. 2022; Wang et al. 2022). Negative controls of IHC staining in db/m and db/db mice were also conducted (Additional file 1: Fig. S2).

### Apoptosis assay

The TUNEL assay was performed according to the manufacturer's instructions (Yang et al. 2019) to evaluate apoptosis in mouse kidney sections. Apoptosis of cells was detected by an Annexin V-FITC Apoptosis detection kit (Beyotime Institute of Biotechnology, China). After the treatments, the cell culture solution was removed, and one PBS wash was performed. Annexin V-FITC binding solution, Annexin V-FITC and PI dye solution were added successively into the cells and mixed gently. After being incubated in the dark for 10–20 min at room temperature (20–25 °C), the cells were placed in an ice bath, observed immediately under a fluorescence microscope (Annexin V-FITC is green fluorescence and PI is red fluorescence), and the integrated optical density of FITC-Annexin V/DAPI was calculated by IPP.

### Oxidative stress assessment

To evaluate tissue ROS levels, we stained kidney slices with dihydroethidium (DHE). DHE powder was dissolved in dimethyl sulfoxide. Cryosections were incubated with the DHE solution at room temperature for 30 min and observed with confocal microscopy. MitoSOX Red was used to measure mitochondrial ROS levels. The cells were incubated with the MitoSOX Red solution for 30 min at 37 °C, then photos were taken under a microscope. And the integrated optical density of DHE/DAPI and MitoSOX/DAPI were calculated by IPP.

### Cell culture

We used HK-2 cells (a human proximal tubular epithelial cell line), which were acquired from ATCC (USA) and cultured in Dulbecco’s modified Eagle’s medium (DMEM)/F12 supplemented with 10% FBS and 100 U/mL penicillin plus 0.1 mg/mL streptomycin for our in vitro studies. Time-dependent experiments were carried out in HK-2 cells that were treated with 30 mM D-glucose for 0–48 h. After the cells had grown to 50–70% confluence, HK-2 cells were transfected with siRNAs or plasmids by Lipofectamine 2000 (Invitrogen, USA) in accordance with the manufacturer’s instructions. Subsequently, after one day of transfection, the cells were stimulated with high glucose (final concentration 30 mmol/L glucose) for 6 h or 24 h and then harvested. The ATF5 siRNA (target sequence: CACCTGACCTGGAAGCTAT) and HSP60 siRNA (target sequence: GGGAAGTCCCAAAGTAACAA) were purchased from RiboBio Co., Ltd. (China), and the pCMV3- hATF5 plasmid was purchased from SinoBio Co., Ltd. (China). Scrambled siRNAs and the empty pCMV3 vector were applied to parallel cultures as negative controls).

### Immunofluorescence analysis

Immunofluorescence (IF) assays were performed to assess the translocation of ATF5. After fixation, infiltration and blocking, HK-2 cells were incubated with anti-ATF5 antibodies at 4 °C overnight. The next day, the cells were incubated with secondary antibodies. Cell nuclei were stained with 4′,6-diamidino-2-phenylindole (DAPI). Images were taken under a confocal microscope.

### Western blotting

Total protein was extracted from mouse kidneys and HK-2 cells in radioimmune-precipitation assay (RIPA) protein lysis buffer according to the protocol. Mitochondrial and nuclear proteins were collected with corresponding kits (Beyotime Institute of Biotechnology, China) according to the manufacturer’s instructions. The concentrations of the extracted proteins were measured by a BCA protein assay kit. SDS loading buffer (5 ×) was added to the samples and boiled for 10 min. Different concentrations of SDS‒PAGE were used to separate the proteins electrophoretically, which were transferred to PVDF membranes. The membranes were incubated with the following primary antibodies overnight at 4 °C: anti-ATF5 (1:1500 dilution, Abcam), anti-HSP60 (1:1000 dilution, Abcam), anti-LONP1 (1:1000 dilution Abcam), anti-fibronectin (1:1000 dilution, Abcam) and anti-C-CAS3 (1:1000 dilution, CST). β-actin (1:5000, Proteintech), VDAC (1:5000, Proteintech) and Lamin B1 (1:5000, Proteintech) were used as internal controls. After treatment with the appropriate secondary antibody, the membranes were observed with enhanced chemiluminescence (ECL, Amersham Pharmacia Biotech, Uppsala, Sweden). The protein levels were analyzed using ImageJ software and normalized to β-actin, VDAC or Lamin B1 levels.

### Statistical analysis

All experiments were repeated at least 3 times. The data were statistically analyzed by SPSS 20.0 software. Values are shown as the mean ± S.D. (standard deviation). The data were analyzed by using one-way ANOVA for multiple groups or Student’s t test for two groups. Pearson’s correlation was used in the correlation analysis. p < 0.05 was considered statistically significant.

## Results

### ATF5 and UPRmt-related molecules were significantly increased in the kidneys of type 2 DKD patients and were positively correlated with tubulointerstitial damage

**Fig. 1:**
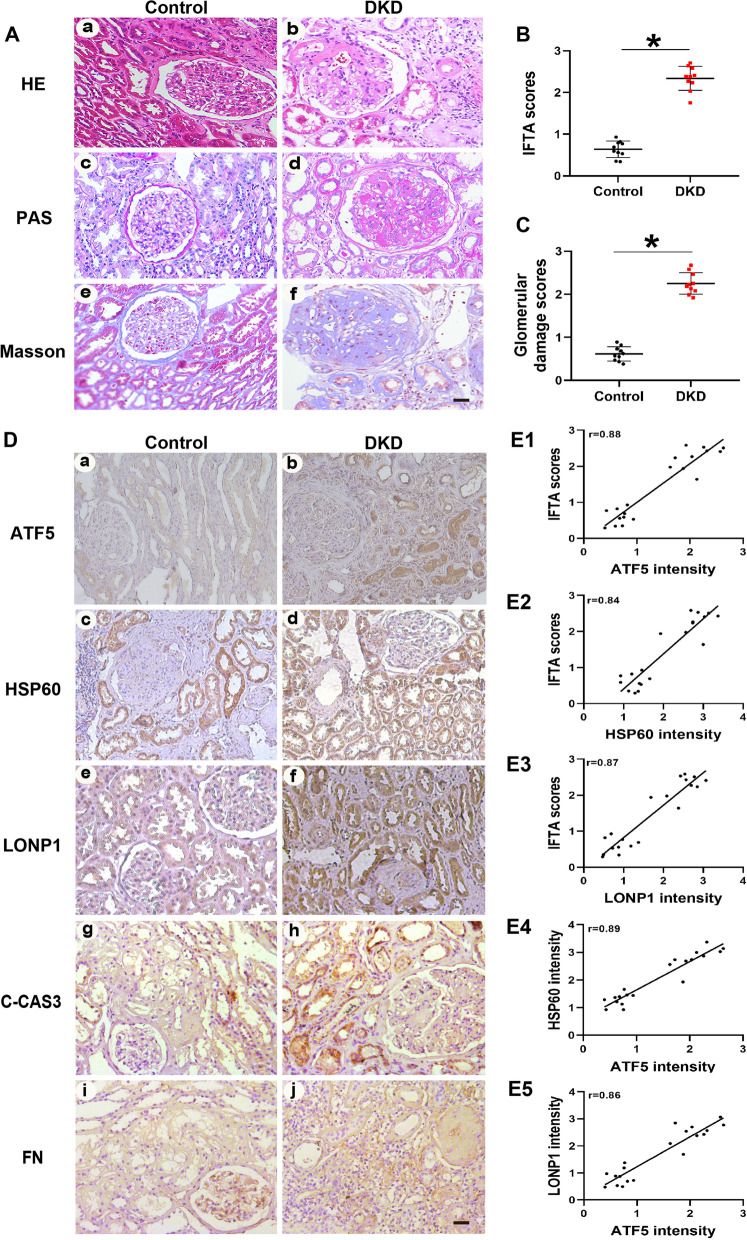
Increased ATF5/HSP60/LONP1 expression positively correlates with tubulointerstitial damage in kidney biopsy sections of DKD patients. **A** HE, PAS and Masson staining of the histological changes in kidney biopsies of DKD patients. **B, C** Quantitative analysis of interstitial fibrosis and tubular atrophy (IFTA) scores and glomerular injury. **D** IHC staining of ATF5/HSP60/LONP1, C-CAS3 and FN in kidney biopsy sections of DKD patients. **E1–E3** The correlations of ATF5, HSP60 and LONP1 expression levels with IFTA scores. **E4–E5** The correlations of HSP60, LONP1 with ATF5 expression in the kidney of type 2 DKD patients. Scale bar: 25 μm. All data are presented as means ± SD; *p < 0 .05 vs control group. r: correlation coefficient. n = 10

To explore the relationship between ATF5 and UPRmt-related proteins in DKD patients, clinical information and tissue sections from 10 patients diagnosed with DKD and 10 patients with minimal glomerular lesions (control group) were collected. The clinical data of the patients are shown in Table 1. As shown by HE, PAS and Masson’s staining, the renal tissues of DKD stage III patients showed mesangial matrix proliferation, tubular atrophy and interstitial fibrosis compared with those of the control group (Fig. 1A). The IFTA scores and glomerular damage scores of patients with DKD were higher than those of the control subjects (Fig. 1B, C). The protein expression of ATF5, HSP60 and LONP1 was increased in the kidneys of DKD stage III patients, especially in the tubulointerstitial area (Fig. 1D). The expression of cleaved caspase 3 (C-CAS3) and fibronectin (FN) was increased in the kidneys of DKD patients (Fig. 1D). Furthermore, there was a strong positive association between the expression of ATF5/HSP60/LONP1 and the tubulointerstitial damage score (Fig. 1E1–3). The expression of ATF5 was positively correlated with the expression of HSP60 and LONP1 (Fig. 1E4, 5). These results indicate that ATF5 is crucial for tubulointerstitial injury in the kidneys of DKD patients through the UPRmt pathway.

**Table 1:** Clinical characteristics of the patients

	Control (n = 10)	DN (n = 10)
Age (y)	33.0 ± 10.4	47.5 ± 6.9
Sex (male/female)	4/6	5/5
Duration (y)	–	13.1 ± 2.9
BMI (kg/m^2^)	22.9 ± 2.6	24.8 ± 3.5
Glucose (mmol/L)	4..2 ± 0.5	7.9 ± 5.3*
Hb (g/L)	32.4 ± 14.3	8.3 ± 1.2
HbA1c (%)	4.7 ± 0.5	7.9 ± 0.5*
Total cholesterol (mmol/L)	4.5 ± 0.9	6.2 ± 0.7
Triglyceride (mmol/L)	1.6 ± 0.5	2.5 ± 0.6
Albumin (g/L)	39.1 ± 4.6	25.2 ± 3.1*
Scr (μmol/L)	66.7 ± 9.3	137.1 ± 17.5*
BUN (mmol/L)	5.1 ± 0.5	9.1 ± 2.3*
UA (μmol /L)	289.3 ± 19.5	365 ± 42.1*
24-h urine protein (g/d)	0.6 ± 0.5	5.1 ± 1.3*
SBP (mm Hg)	108 ± 6.7	119 ± 8.2
DBP (mm Hg)	69 ± 8.5	82 ± 11.3

*BMI* body mass index, *BUN* blood urea nitrogen, *DBP* diastolic blood pressure, *Hb* hemoglobin, *HbA1c* glycosylated hemoglobin, *SBP* systolic blood pressure, *Scr* serum creatinine, *UA* uric acid. *p < 0.5 vs control; values are Mean ± SD

### The expression of ATF5 in the kidneys of db/db mice was positively associated with the level of UPRmt-related molecules

**Fig. 2:**
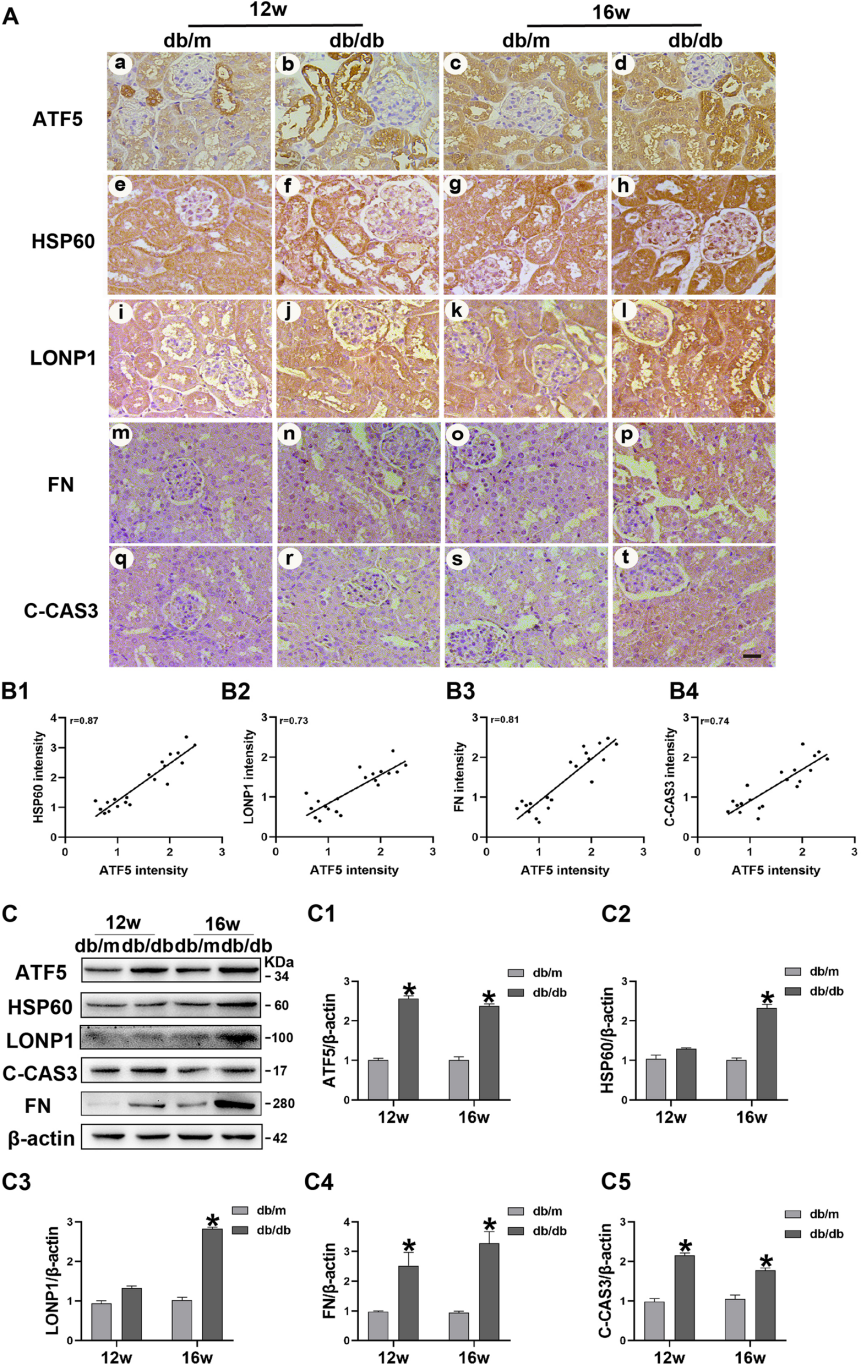
Increased ATF5/HSP60/LONP1 expression in the kidneys of db/db mice and the correlation with tubular damage. **A** IHC staining of ATF5, HSP60, LONP1, FN and C-CAS3 in the kidney sections of 12/16-week-old db/db mice. Scale bar: 30 μm. **B1–B4** The correlations of HSP60, LONP1, FN, C-CAS3 with ATF5 expression in the kidney of db/db mice. **C** Western blot analysis of ATF5 and HSP60, LONP1, FN, C-CAS3 in mice kidney tissue. **C1–C5** Relative band density. All data are presented as means ± SD; *p < 0.05 vs db/m. r: correlation coefficient. n = 6

IHC staining showed that the intensity of ATF5, HSP60, LONP1, FN and C-CAS3 was increased in the renal tubules of 12/16-week-old db/db mice compared with those in the control group (Fig. 2A). Moreover, the expression of ATF5 was positively associated with the expression of HSP60, LONP1, FN and C-CAS3 (Fig. 2B1–4). These changes were further confirmed by western blot analysis and semiquantitative analysis (Fig. 2C, C1–5). These results suggest that ATF5 may be involved in the progression of tubulointerstitial injury in the kidneys of db/db mice via the UPRmt pathway.

### Knockdown of ATF5 conferred renal protection against apoptosis and oxidative stress and reduced the expression of UPRmt-related proteins in db/db mice

**Fig. 3:**
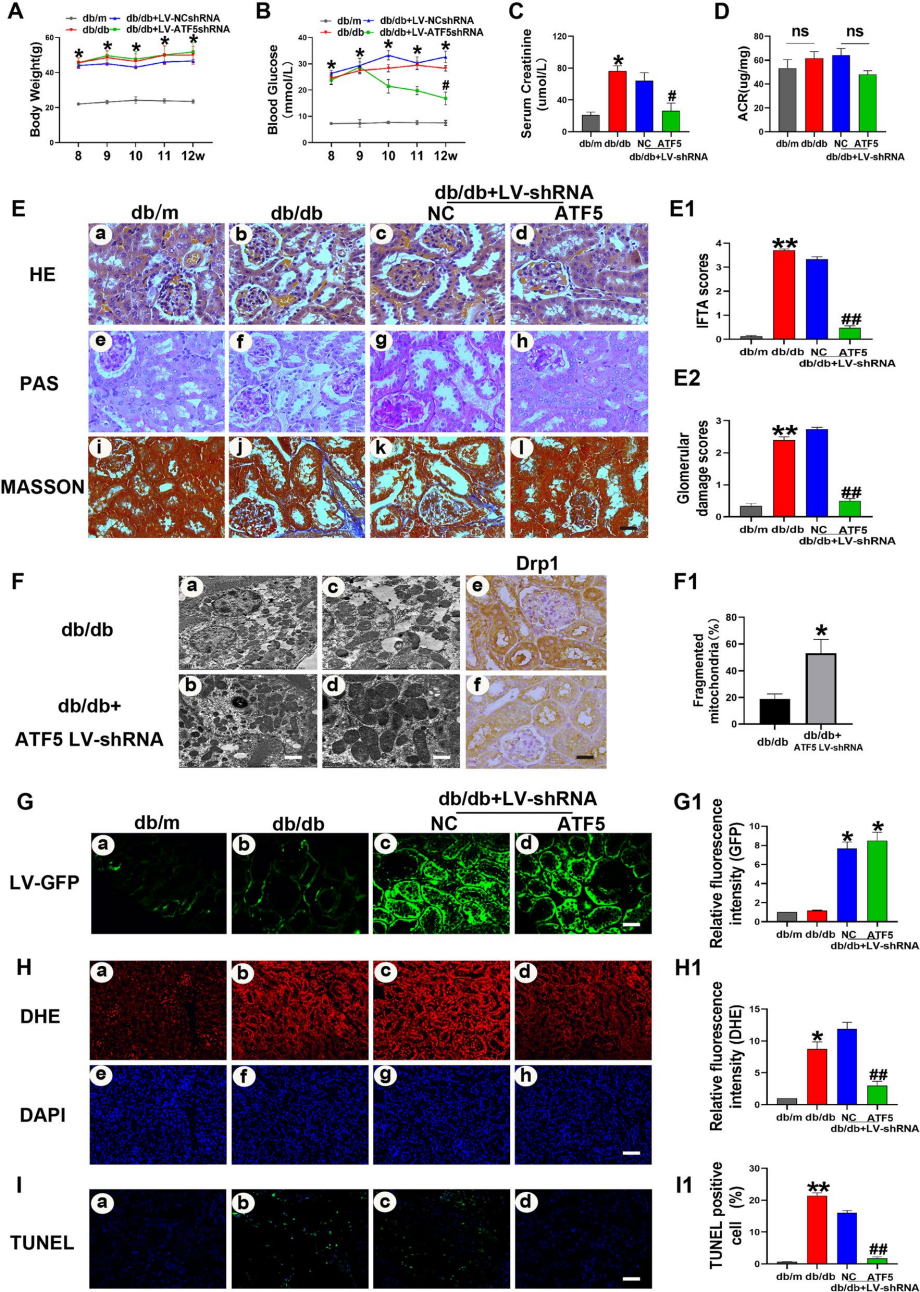
ATF5 downregulation ameliorates tubulointerstitial injury in db/db mice. **A** Bodyweight changes. **B** Blood glucose concentrations. **C** Serum creatinine level. **D** Urinary albumin excretion (ACR). **E** Morphological examinations of tubular and glomerular changes by HE, PAS, Masson. Scale bars: 30 μm. **E1–E2** Quantitative analysis of interstitial fibrosis and tubular atrophy (IFTA) scores, glomerular damage in the kidneys. **F** The thickness of the basement membrane and mitochondrial fragmentation in renal tissue are shown by transmission electron microscopy (TEM) (**a–d**). Lower scale bar: 2.5 μm, upper scale bar: 1 μm; IHC images of Drp1 in mice kidney (**e**, **f**). Scale bar: 30 μm. **G** GFP fluorescence images in kidney tissues of mice. Scale bar: 30 μm. **H** the production of superoxide was assessed by DHE. Scale bar: 50 μm. **I** TUNEL staining in renal tissues of mice. Scale bar: 50 μm. **G1–I1** Semiquantification of GFP and DHE fluorescence density and statistical analysis of TUNEL-positive cells, the integrated optical density of DHE/DAPI was calculated by IPP. All data are presented as means ± SD; *p < 0.05, **p < 0.01 vs. db/m; ^#^p < 0.05, ^##^p < 0.01 vs. db/db + LV-NC-shRNA; ns, no statistical significance. n = 6

**Fig. 4:**
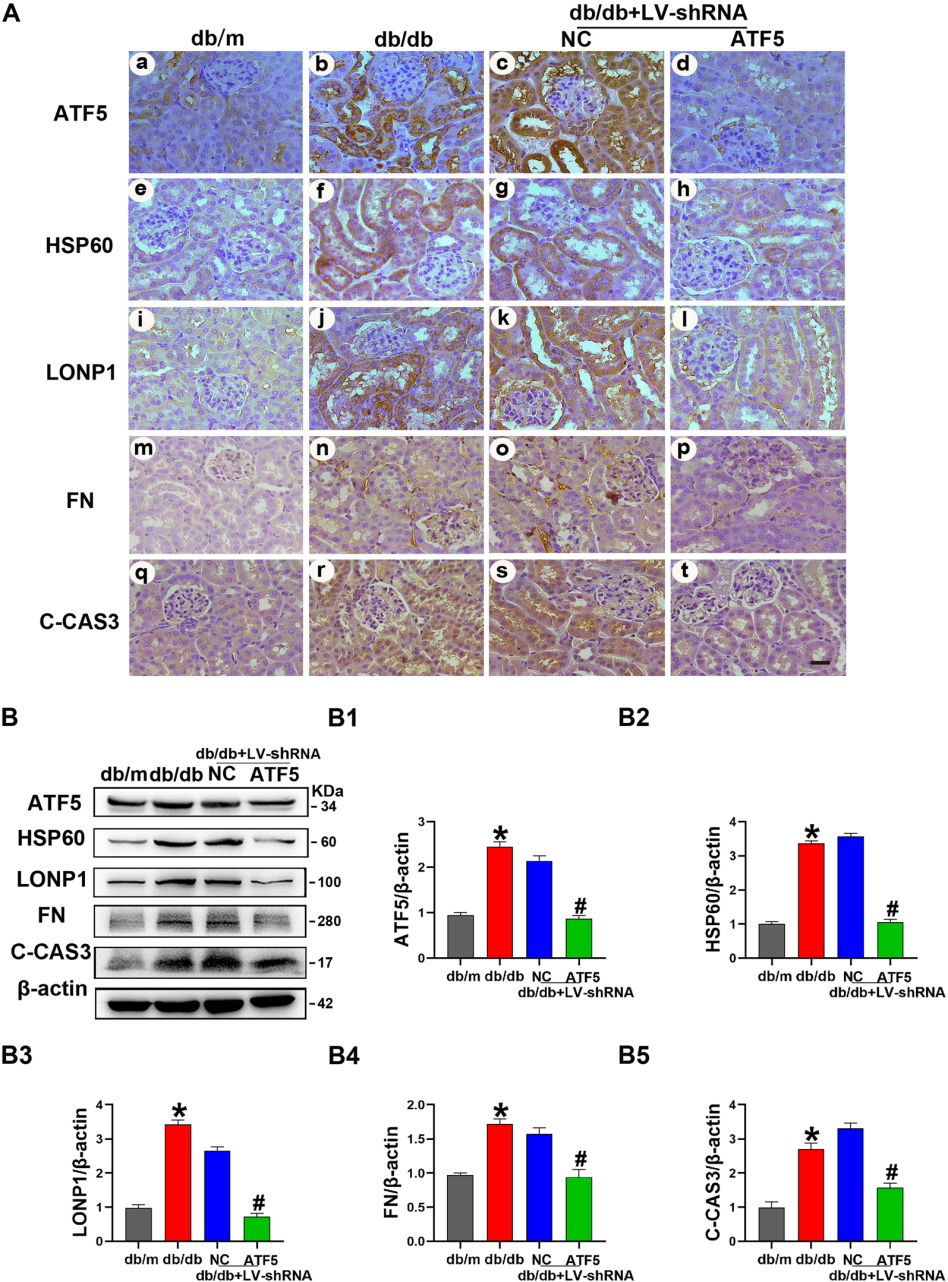
Upregulated expression of ATF5/HSP60/LONP1, FN/C-CAS3 in the kidney sections of db/db mice was attenuated by injection of ATF5 LV-shRNA. **A** Representative IHC images of ATF5, HSP60, LONP1, FN, C-CAS3 in the kidneys of mice. Scale bar: 30 μm. **B** Western blot analysis of ATF5, HSP60, LONP1, FN, C-CAS3. **B1–B5** Relative band density of ATF5, HSP60, LONP1, FN, C-CAS3. All data are presented as means ± SD; *p < 0.05 vs. db/m; ^#^p < 0.05 vs. db/db + LV-NC-shRNA. n = 6

To determine the specific role of ATF5 in tubulointerstitial injury in vivo, eight-week-old db/db mice were injected with ATF5-shRNA lentiviruses via the tail vein, and a negative lentivirus was used as a control. Blood glucose and serum creatinine levels were significantly decreased in the db/db + LV-shRNA-ATF5 group compared to the db/db + LV-NC group at week 12 (Fig. 3B, C). There were no statistically significant differences in body weight or ACR between the two groups (Fig. 3A and D). Morphology alterations, the thickness of the basement membrane and mitochondrial fragmentation in renal tissue were exacerbated in db/db mice, and these effects were dramatically alleviated in db/db mice injected with ATF5-shRNA lentivirus (Fig. 3E, F, a–d). Semiquantitative analysis of interstitial fibrosis and tubular atrophy (IFTA) scores, glomerular injury and Drp1 protein expression verified these results (Fig. 3E1, E2, F, e–f). GFP fluorescence images confirmed the successful viral infection of kidney tissues in the LV-shRNA injected groups (Fig. 3G). DHE and TUNEL staining revealed that ATF5 LV-shRNA transfection notably reduced superoxide production and TUNEL-positive cells in db/db + LV shRNA-ATF5 mice (Fig. 3H, I). Moreover, the IHC results revealed a significant increase in the expression of ATF5, HSP60, LONP1, FN and C-CAS3 in db/db and db/db + LV-NC mice compared with db/m mice, especially in the renal proximal tubules, while the expression of these proteins was notably inhibited in db/db + LV-shRNA-ATF5 mice (Fig. 4A). A similar phenomenon was confirmed by western blot analysis (Fig. 4B). These results demonstrated that knockdown of ATF5 in the kidneys of db/db mice prevented tubular oxidative stress and apoptosis by regulating the UPRmt pathway.

### The upregulation of ATF5 and UPRmt-related proteins in HK-2 cells induced by high glucose (30 mM) was time dependent

**Fig. 5:**
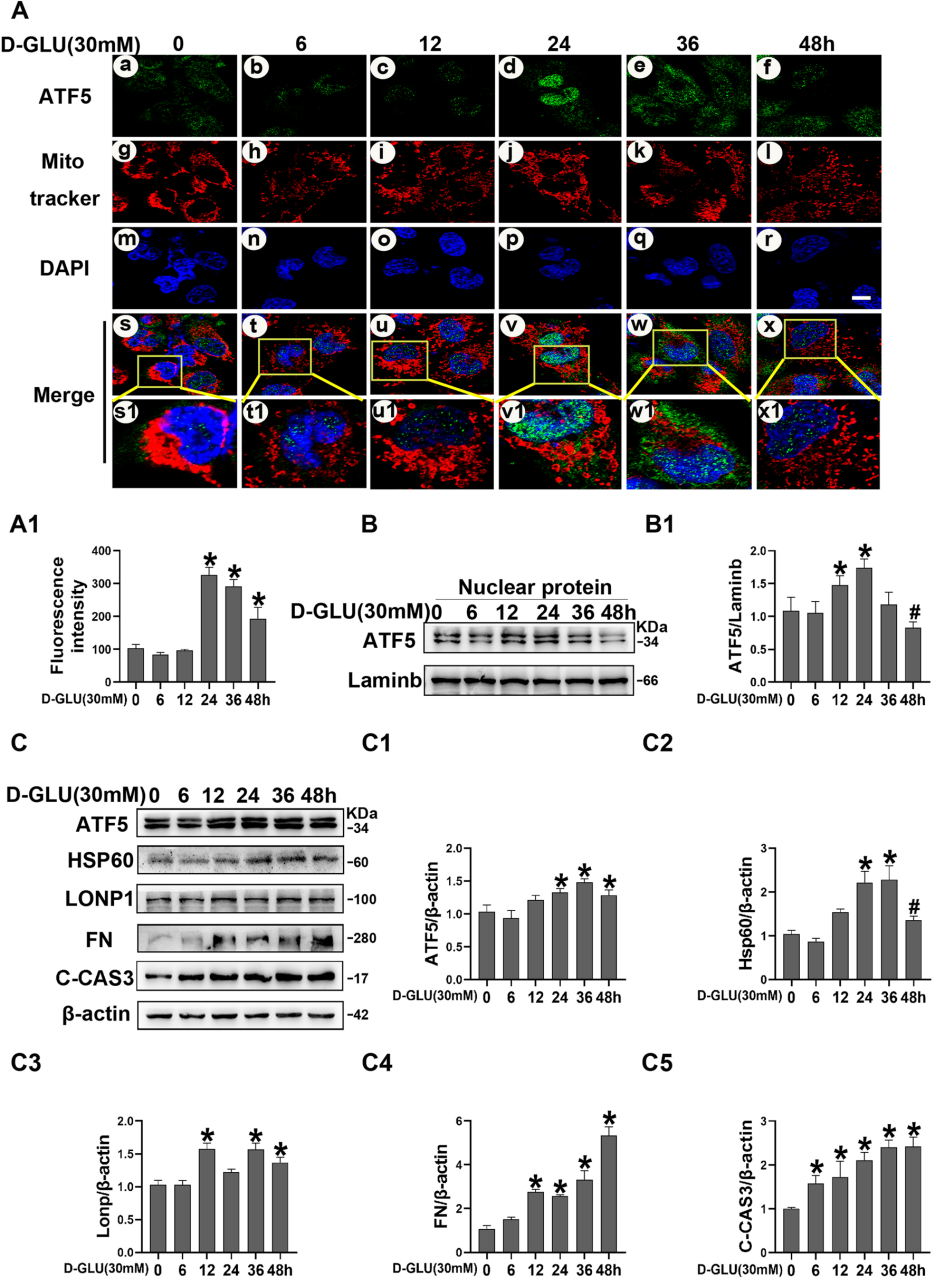
ATF5 and UPRmt related molecule expression in HK-2 cells incubated with HG for 0–48 h. **A** Immunofluorescence analysis were performed to access ATF5 nuclear translocation in HK-2 cells. Scale bar: 2 μm. **A1** Semiquantification of green fluorescence density of ATF5. **B** Western blot analysis of ATF5 nuclear protein expression in HK-2 cells. **B1** Relative band density of ATF5. **C** Western blot analysis of ATF5, HSP60, LONP1, FN, C-CAS3 expression in HK-2 cells with 30 mM HG exposure for 0–48 h. **C1–C5** Relative band density. All data are presented as means ± SD; *p < 0.05 vs LG; ^#^p < 0.05 vs 36 h HG. n = 3

To further investigate the effect of HG on the expression of ATF5 and UPRmt-related proteins, HK-2 cells were cultured in 30 mM HG for 0, 6, 12, 24, 36 and 48 h. Since ATF5 is thought to activate UPRmt transcription through nuclear translocation, ATF5 expression was examined under an immunofluorescence microscope. As shown in Fig. 5A, HG induced the expression of ATF5 and slight nuclear translocation at 6 h and 12 h, which peaked at 24 h, started to decrease at 36 h and decreased to control levels at 48 h (Fig. 5A). This time-dependent ATF5 nuclear translocation was confirmed via western blotting (Fig. 5B). On the other hand, proteins extracted from whole cell lysates revealed that ATF5 and HSP60 expression began to increase after exposure to high glucose for 12 h, peaked at 36 h and slightly decreased at 48 h. For LONP1, cytoplasmic expression remained elevated from 12 to 48 h, although at 24 h, the expression was lower than that at 12 h, 36 h and 48 h. In addition, a significant time-dependent increase in cytoplasmic FN and C-CAS3 was observed (Fig. 5C).

### Knockdown of ATF5 exacerbated oxidative stress and apoptosis in HK-2 cells during the early stage of HG incubation (6 h)

**Fig. 6:**
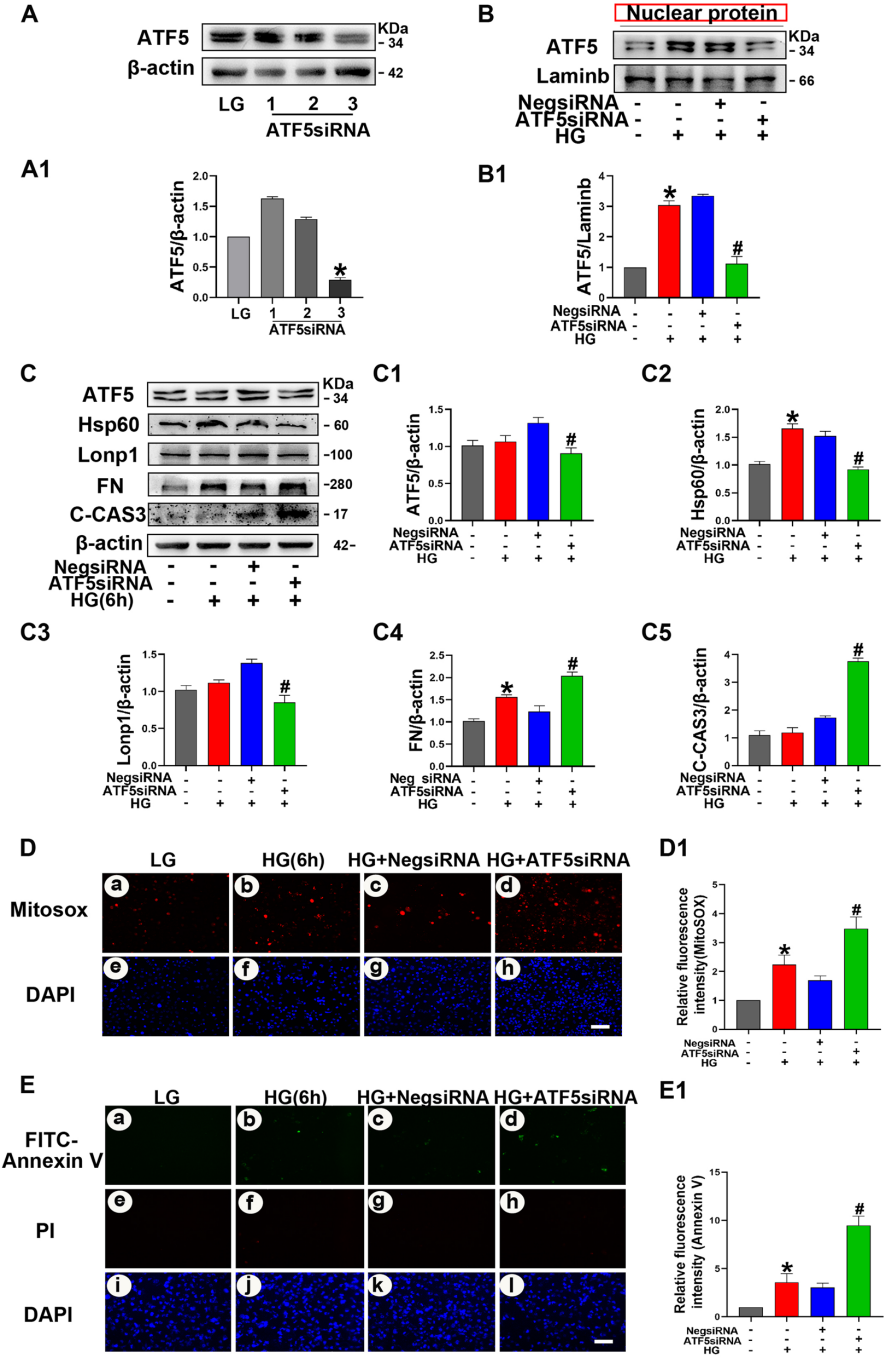
Knockdown of ATF5 downregulates the HSP60/LONP1 expression, exacerbated injury in HK-2 cells after 6 h HG exposure. **A** The efficiency of ATF5 siRNA3-mediated knockdown was verified by Western blot analysis. **A1** Relative band density of ATF5. **B** Western blot analysis of ATF5 nuclear protein in HK-2 cells. **B1** Relative band density. **C** Western blot analysis of ATF5, HSP60, LONP1, FN, C-CAS3 cellular proteins after transfecting ATF5-siRNA in HK-2 cells treated with HG for 6 h. **C1–C5** Relative band density. **D** MitoSOX dye was used to detect mitochondrial ROS. Scale bar: 30 μm. **E** Representative microscopy images of FITC-Annexin staining. Scale bar: 30 μm. **D1–E1** Semiquantification of Relative fluorescence intensity, the integrated optical density of MitoSOX/DAPI and FITC-Annexin V/DAPI were calculated by IPP. All data are presented as means ± SD; *p < 0.05 vs LG; ^#^p < 0.05 vs 6 h HG + Neg-siRNA. n = 3

To observe the effect of ATF5 on HK-2 cells during the early stage of HG treatment, we used ATF5 siRNA to knock down ATF5 expression in HK-2 cells. The efficiency of ATF5 siRNA3-mediated knockdown was verified, and it efficiently inhibited ATF5 nuclear protein expression in HK-2 cells cultured with HG (Fig. 6A and B). HK-2 cells were transfected with ATF5 siRNA for 24 h and then treated with 6 h HG. Western blotting showed a significant decrease in the expression of HSP60 and LONP1, while the expression of FN and C-CAS3 increased dramatically (Fig. 6C). Mitochondrial ROS levels and cell apoptosis were correspondingly increased (Fig. 6D, E). These data suggested that ATF5 may play a protective role in HK-2 cells during the early stage of HG exposure.

### Knockdown of ATF5 in HK-2 cells reversed oxidative stress and apoptosis induced by continuous high glucose exposure

**Fig. 7:**
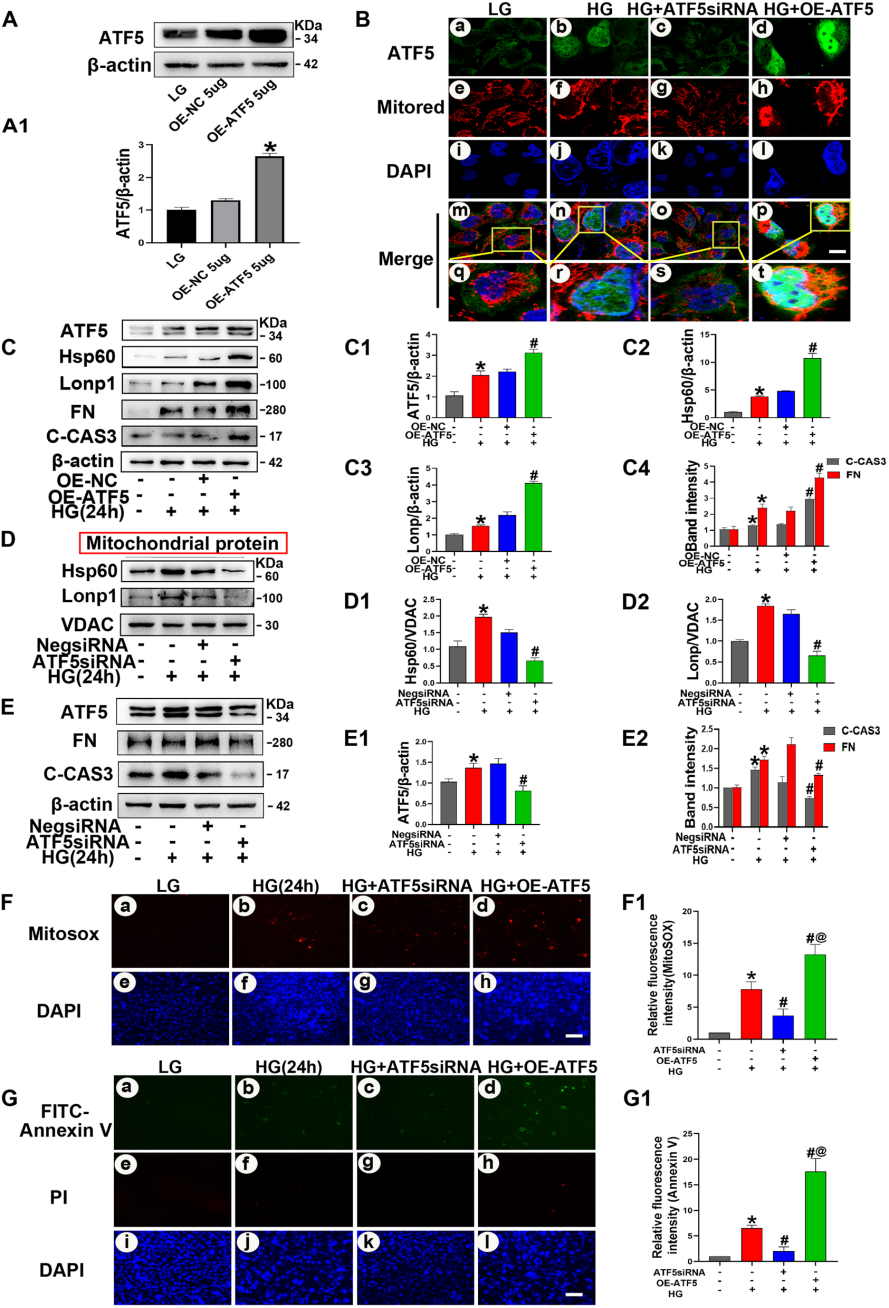
ATF5 knockdown reversed damage induced by continuous HG exposure in HK-2 cells, while overexpression ATF5 exacerbated these effect. **A** Western blot analysis to confirm ATF5 plasmid transfected efficiency. **A1** Relative band density of ATF5. **B** Immunofluorescence analysis were performed to access ATF5 nuclear translocation in HK-2 cells. Scale bar: 2 μm. **C** Western blot analysis of ATF5, HSP60, LONP1, FN, C-CAS3 expression in whole cell lysate. **C1–C4** Relative band density. **D** Western blot analysis of HSP60, LONP1 mitochondrial proteins. **D1–D2** Relative band density. **E** Western blot analysis of ATF5, FN, C-CAS3 in whole cell lysate. **E1–E2** Relative band density. **F** MitoSOX dye was used to detect mitochondrial ROS. Scale bar: 30 μm. **G** Representative microscopy images of FITC-Annexin staining. Scale bar: 30 μm. **F1–G1** Semiquantification of Relative fluorescence intensity, the integrated optical density of MitoSOX/DAPI and FITC-Annexin V/DAPI were calculated by IPP. All data are presented as means ± SD; *p < 0.05 vs LG; ^#^p < 0.05 vs 24 h HG. ^@^p < 0.05 vs 24 h HG + ATF5-siRNA. n = 3

To determine the effect of ATF5 on HK-2 cells under continuous high-glucose stimulation, HK-2 cells were transfected with ATF5 siRNA and then treated with HG for 24 h. Immunofluorescence analysis revealed that compared with that in the LG group, ATF5 expression was increased in HK-2 cells in the HG and HG + Neg siRNA groups, especially in the nucleus, while ATF5 expression was significantly decreased with nearly no nuclear translocation in the HG + ATF5 siRNA group (Fig. 7B). Given that HSP60 and LONP1 are located in mitochondria, mitochondrial proteins were extracted under the same treatment conditions. As expected, ATF5 knockdown decreased HSP60 and LONP1 expression (Fig. 7D). Contrary to the early stage of HG intervention, western blotting showed that ATF5 knockdown led to a notable decrease in FN and C-CAS3 protein expression (Fig. 7E) and decreased the levels of mitochondrial ROS and cell apoptosis (Fig. 7F, G). These results indicated that ATF5 exacerbated the damage caused by continuous HG stimulation in HK-2 cells by upregulating the UPRmt.

### ATF5 overexpression exacerbated fibrosis, apoptosis and oxidative stress in HK-2 cells treated with 30 mM HG for 24 h

To further determine the role of ATF5 in HK-2 cells with continuous HG stimulation, ATF5 was overexpressed by the ATF5 plasmid. Western blotting verified the transfection efficiency of the ATF5 plasmid (Fig. 7A). Compared with those in the HG group, ATF5 expression and nuclear translocation were notably upregulated in the OE-ATF5 group, as shown by immunofluorescence microscopy (Fig. 7B). Compared to those in the HG + OE-NC group, the protein expression of HSP60, LONP1, FN, and C-CAS3 and mitochondrial ROS production, as well as early apoptosis levels, were dramatically increased in the HG + OE-ATF5 group (Fig. 7C, F, G). These findings further confirmed the role of ATF5 in the damage caused by continuous HG stimulation in HK-2 cells (Fig. 9).

### ATF5 plasmids and HSP60-siRNA transfection blocked the negative effect of ATF5 on HK-2 cells with continuous HG treatment

**Fig. 8:**
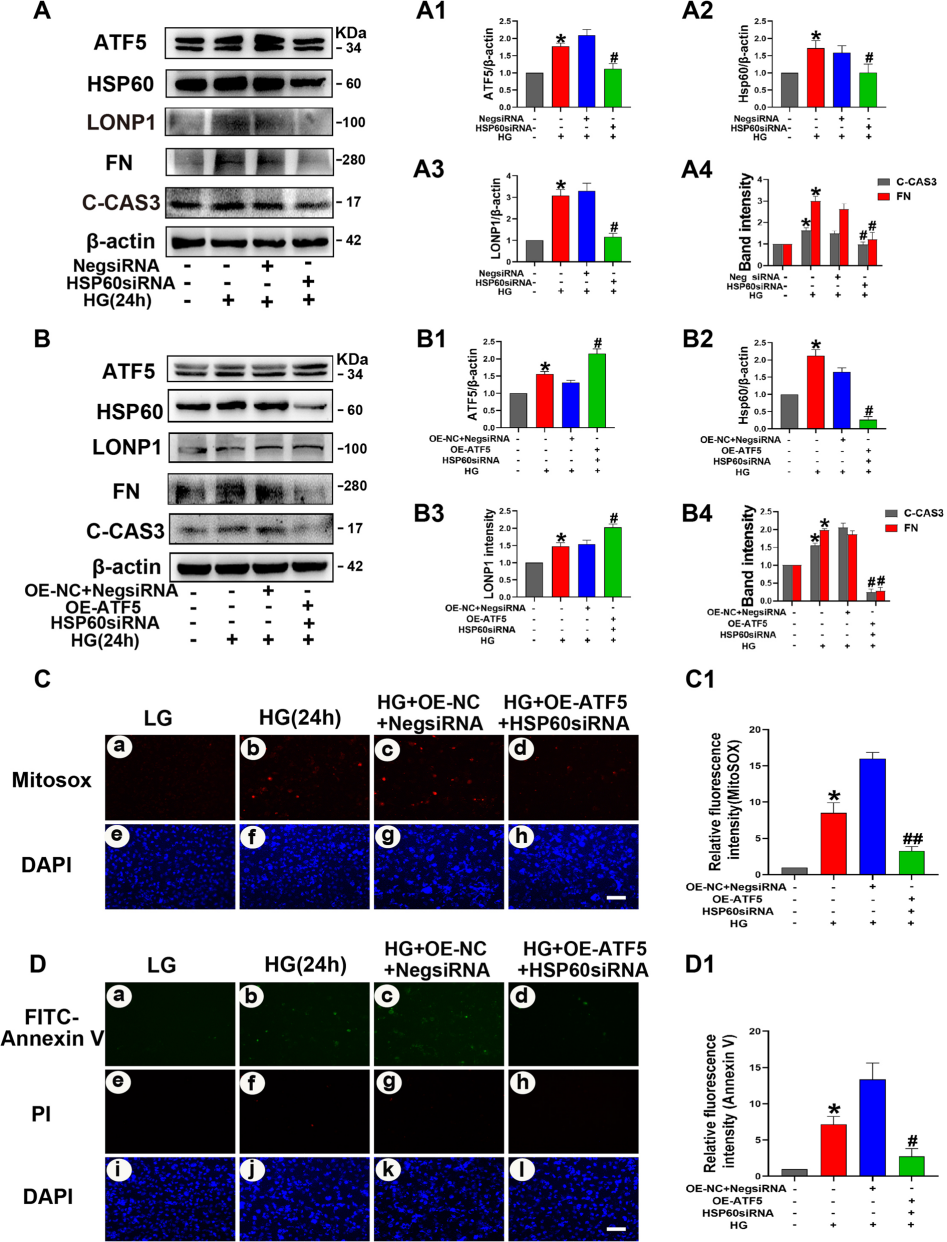
ATF5 plasmids and HSP60-siRNA transfection blocked the negative effect of ATF5 on HK-2 cells with continuous HG treatment. **A** Western blot analysis of ATF5, HSP60, LONP1, FN, C-CAS3 expression in HK-2 cells transfected with HSP60-siRNA alone. **A1** Relative band density. **B** Western blot analysis of ATF5, HSP60, LONP1, FN, C-CAS3 expression in HK-2 cells transfected with ATF5 plasmid and HSP60-siRNA. **B1** Relative band density. **C** MitoSOX dye was used to detect mitochondrial ROS. Scale bar: 30 μm. **D** Representative microscopy images of FITC-Annexin staining. Scale bar: 30 μm. **C1–D1** Semiquantification of Relative fluorescence intensity, the integrated optical density of MitoSOX/DAPI and FITC-Annexin V/DAPI were calculated by IPP. All data are presented as means ± SD; *p < 0.05 vs LG; ^#^p < 0.05, ^##^p < 0.01 vs 24 h HG + Neg-siRNA + OE-NC. n = 3

To investigate whether ATF5 functions through the UPRmt pathway, HK-2 cells were transfected with HSP60-siRNA. HSP60 knockdown significantly decreased the protein expression of ATF5, HSP60, LONP1, FN and C-CAS3 (Fig. 8A). Subsequently, the ATF5 plasmid and HSP60-siRNA were transfected into HK-2 cells in parallel. Compared with that in the negative control group, ATF5 expression increased significantly, while the expression of HSP60, LONP1, C-CAS3, and FN was markedly decreased in the cotransfection group after treatment with 30 mM HG for 24 h (Fig. 8B). Fluorescence staining showed reductions in mitochondrial ROS and apoptosis in HK-2 cells were observed in the cotransfection group (Fig. 8C, D). Overall, these data suggest that HSP60 is a key modulator of ATF5-activated UPRmt in HK-2 cells cultured with continuous high glucose.

## Discussion

The present study demonstrated that ATF5 plays a crucial role in tubulointerstitial injury in DKD, especially in modulating oxidative stress and apoptosis. Reduced mitochondrial ROS production, downregulated expression of UPRmt-related proteins (such as HSP60 and LONP1), FN and C-CAS3 and the amelioration of tubulointerstitial fibrosis and tubular atrophy were observed in db/db mice injected with the ATF5-shRNA lentivirus. In addition, ATF5-siRNA transfection inhibited the expression of UPRmt-related proteins such as HSP60 and LONP1, which was accompanied by reduced oxidative stress and apoptosis in HK-2 cells exposed to sustained exogenous high glucose. ATF5 overexpression exacerbated these impairments. Further experiments showed that transfection with HSP60-siRNA could block these effect of ATF5 on HK-2 cells exposed to continuous HG treatment. Interestingly, ATF5 inhibition exacerbated mitochondrial ROS production and apoptosis in HK-2 cells on the early period of HG intervention (6 h). Overall, ATF5 promoted tubulointerstitial injury under long-term hyperglycemia by regulating the excessive UPRmt pathway, although it might exert a protective effect in the very early stage.

The UPRmt was first proposed in mammalian cells in 1996 and is a key process involved in mitochondrial protein synthesis, folding and degradation (Martinus et al. 1996). Increased expression of chaperone proteins (HSP60 and HSP10) and mitochondrial proteases (ClpP and LONP1) during the UPRmt could modulate MQC and balance mitochondrial protein dynamics, thus modulating mitochondrial function (Voos et al. 2016). Zhao Q et al. observed the aggregation of mitochondrial matrix proteins and an increase in HSP60 and ClpP transcription in COS-7 cells transfected with OTC-Δ to activate the UPRmt response (Zhao et al. 2002). Many studies have suggested that the UPRmt is closely correlated with development, aging, innate immunity, erythrocyte differentiation, tumor pathogenesis, bacterial infection and metabolic disorders (Martinus et al. 1996; Pellegrino and Haynes 2015; Shpilka and Haynes 2018; Fiorese and Haynes 2017; Picca et al. 2018). Increased expression of HSP60, HSP10, LONP1 and ClpP protein was observed in the liver in an HFD-induced type 2 diabetes mouse model (Wu et al. 2014). The present study demonstrated that the UPRmt was activated in the kidney, especially in the renal tubules of DKD patients and db/db mice compared with controls, and this effect was positively correlated with interstitial fibrosis and tubular atrophy (IFTA) (Figs. 1 and 2). However, the underlying mechanism remains to be elucidated.

ATF5 is a downstream target gene of pancreatic and duodenal homeobox 1 (Pdx1), which is a key pathogenetic factor of diabetes. Fatty acids induce ATF5 overexpression, while a lack of Pdx1 inhibits ATF5 transcription in pancreatic β cells. Moreover, knockdown of ATF5 alleviates β cell apoptosis induced by various stresses (Juliana et al. 2017). Importantly, ATF5 is a critical regulator of the UPRmt response. A marked increase in ATF5 transcription was observed in HEK293T cells treated with paraquat and OTC-Δ (Deng and Haynes 2017). ATF5 can modulate the expression of ClpP and HSP60, leading to tumor cell apoptosis and cell growth (Fiorese et al. 2016). Moreover, it was suggested that inhibiting mitochondrial respiratory chain complexes I and III increased LONP1 mRNA levels in HEK293T cells in an ATF5-dependent manner (Fiorese et al. 2016). In this study, ATF5 expression was dramatically increased in the kidney, particularly in the proximal tubules of DKD patients and db/db mice compared with the control, and this change was positively correlated with HSP60, LONP1 and IFTA levels (Figs. 1 and 2). In addition, ATF5-shRNA lentivirus injection significantly abolished mitochondrial oxidative stress products and tubular cell apoptosis, as shown by reductions in blood glucose and serum creatinine levels and the amelioration of tubulointerstitial fibrosis in db/db mice (Fig. 3). These results indicate that ATF5 modulates MQC and contributes to tubular injury under DKD conditions.

The UPRmt is initiated by mild mitochondrial stress and is an adaptive mechanism that can promote mitochondrial function repair (Czajka and Malik 2016; Covington and Schnellmann 2012). Ghrelin treatment upregulated HSP60 and HSP10 expression and prevented brain mitochondrial dysfunction after cardiac arrest (Xu et al. 2019). LONP1 protected against TDP-43-induced neurodegeneration in Drosophila in the early stage (Wang et al. 2019). Moreover, nicotinamide riboside was shown to activate the UPRmt and abate the reduction in mitochondrial oxygen consumption caused by stress in cardiomyocytes (Smyrnias et al. 2019). In our study, HSP60 and LONP1 expression was increased in the kidney tissue of DKD patients and db/db mice but was inhibited by ATF5 knockdown in db/db mice (Figs. 1, 2 and 4). Subsequent in vitro experiments showed that ATF5 was increased in HK-2 cells exposed to high glucose (HG) in a time-dependent manner, translocated to the nucleus as early as 6 h, and peaked at 24 h (Fig. 5), as previously reported (Fiorese et al. 2016). In addition, the expression of HSP60 and LONP1 was increased in HK-2 cells treated with HG (Fig. 5). Knockdown of ATF5 inhibited HSP60 and LONP expression and increased mitochondrial ROS production and tubular cell apoptosis after 6 h of exposure to HG (Fig. 6). These results suggest that ATF5 coordinates with the UPRmt to play a protective role against tubular cell damage in the early stage of HG exposure.

Emerging evidence suggests that prolonged activation or dysregulation of the UPRmt has a negative impact. Persistent stress can induce excessive UPRmt activation, which leads to mitochondrial lesions and abnormal proteomes, such as PINK accumulation and aberrant mitophagy levels, which can even initiate apoptosis (Pellegrino and Haynes 2015; Deng and Haynes 2017). Inhibiting overwhelming UPRmt activation with rapamycin could decrease deleterious mtDNA levels and slow mitochondrial myopathy progression (Burbulla et al. 2014). It was suggested that in the initial stages of diabetes, proteotoxic mitochondria stress in the brain was stringently controlled by mitochondrial proteases and chaperonins (HSP, HSC20, TID1) (Yi et al. 2018). Persistent oxidative stress due to chronic hyperglycemia results in protein aggregate accumulation and deficient protein quality control in the diabetic brain (Fernandes et al. 2020). Similarly, in contrast to the protective effect in the early stage, knockdown of ATF5 protected against tubular cell injury after 24 h of exposure to HG, while ATF5 overexpression exacerbated tubular injury (Fig. 7). Combining the restoration of mitochondrial morphology and the amelioration of ROS production, apoptosis and tubulointerstitial fibrosis with ATF5 lentivirus shRNA treatment in db/db mice (Figs. 3 and 4), these data indicate that ATF5 induces continuous UPRmt activation, which increases tubular cell injury and leads to tubulointerstitial fibrosis under sustained HG exposure.

**Fig. 9:**
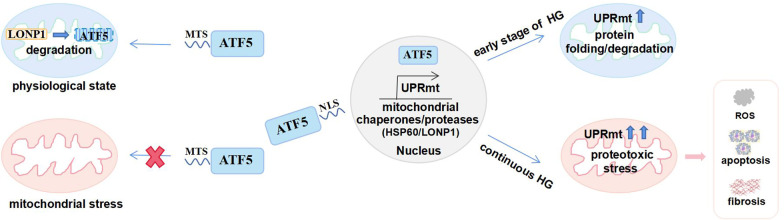
Schematic diagram depicting the mechanism of which ATF5-modulated UPRmt in the tubular injury under diabetic conditions. ATF5 coordinates with the UPRmt to play a protective role against tubular cell damage in the early stage of HG exposure (6 h), while ATF5 induces continuous UPRmt activation, which increases tubular cell injury and leads to tubulointerstitial fibrosis under sustained HG exposure. *ATF5* activating transcription factor 5, *UPRmt* mitochondrial unfolded protein response, *HSP60* heat shock protein 60, *LONP1* Lon peptidase 1, mitochondrial, *HG* high glucose, *MTS* mitochondrial targeting sequence, *NLS* nuclear localization sequence

HSP60 is widely thought to be associated with cell stress (Macario and Conway de Macario 2007), and regulates protein folding, accumulation and apoptosis (Saibil 2013; Kirchhoff et al. 2002; Chandra et al. 2007). In addition, it was demonstrated that HSP60 could maintain mitochondrial morphology and control mitochondrial activity (Veereshwarayya et al. 2006; Lin et al. 2001; Magnoni et al. 2013). Proteomics analysis of DKD-related factors showed that HSP60 was closely associated with DKD. The increased expression of HSP60 precedes the occurrence of hyperplasia and apoptosis (Aluksanasuwan et al. 2017b). Moreover, HSP60 plays a key role in oxidative stress, ATP generation, and apoptosis under DKD conditions (Aluksanasuwan et al. 2017a). In the present study, HSP60-siRNA transfection significantly inhibited HG-induced overexpression of ATF5, LONP1, FN, and C-CAS3 in HK-2 cells. Cotransfection of HSP60-siRNA and the OE-ATF5 plasmid offset the hastening effect of transfecting the ATF5 plasmid alone in HK-2 cells under hyperglycemic conditions (Fig. 8). These results indicate that HSP60 is a key modulator of ATF5-induced UPRmt and tubulointerstitial injury in DKD (Fig. 9).

## Conclusions

In conclusion, this study showed that ATF5 plays a prominent role in oxidative stress and apoptosis in tubule cells in DKD. The mechanism might involve excessive UPRmt activation, which provides a novel therapeutic target to ameliorate tubular injury in DKD.

## Supplementary Information

Additional file 1: Figure S1. Transduction efficiency of the Bumpt cellstransfected with LV-ATF5-shRNA. GFP fluorescence imagesand western blotting of ATF5 expressionin Bumpt cells. All data are presented as means ± SD; * p < 0.05 vs LV-NC-shRNA. n = 3. Figure S2. Representative IHC images of negative controland antibody staining. HSP60, LONP1and their negative control of IHC staining in db/m and db/db mice.

## Data Availability

The data sets used and/or analyzed during the current study are available from the corresponding author on reasonable request.

## Abbreviations

MQC: Mitochondrial quality control
DKD: Diabetic kidney disease
UPRmt: Mitochondrial unfolded protein response
ATF5: Activating transcription factor 5
HSP60: Heat shock protein 60
HSP10: Heat shock protein 10
LONP1: Lon peptidase 1, mitochondrial
ClpP: Caseinolytic protease
ATFS-1: Activating transcription factor associated with stress-1
MTS: Mitochondrial targeting sequence
NLS: Nuclear localization sequence
ROS: Reactive oxygen species
FN: Fibronectin
C-CAS3: Cleaved-caspase3
HG: High glucose
Pdx1: Pancreatic duodenal homeobox-1 (Pdx1)
UACR: Urinary albumin‐creatinine ratio
T2DM: Type 2 diabetes mellitus patients
HK-2 cells: Human proximal tubular epithelial cell line
HE: Hematoxylin–eosin
PAS: Periodic acid-Schiff
Masson: Masson trichrome staining
DHE: Dihydroethidium
TUNEL: TdT-mediated dUTP nick end labeling
IHC: Immunohistochemistry
MitoSOX: Mitochondrial Superoxide Indicator
