# Supplementary material for: ATF5 regulates tubulointerstitial injury in diabetic kidney disease via mitochondrial unfolded protein response
Source: Mol Med. 2023 Apr 24;29:57. doi: 10.1186/s10020-023-00651-4 (PMC10127323; doi:10.1186/s10020-023-00651-4)

Figure S1 Transduction efficiency of the Bumpt cells (mouse renal tubular epithelial cells) transfected with LV-ATF5-shRNA.

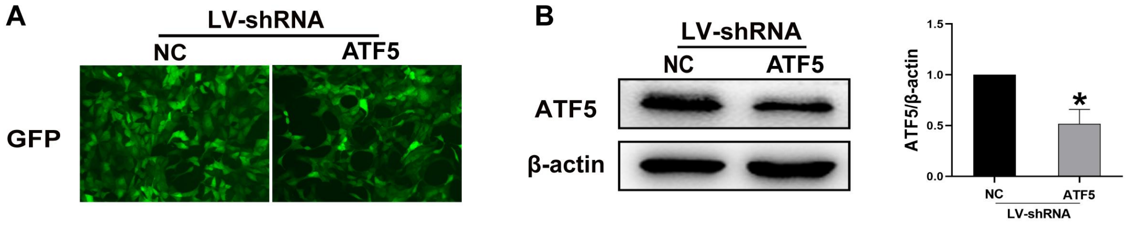

Figure S2 Representative IHC images of negative control (PBS) and antibody staining.

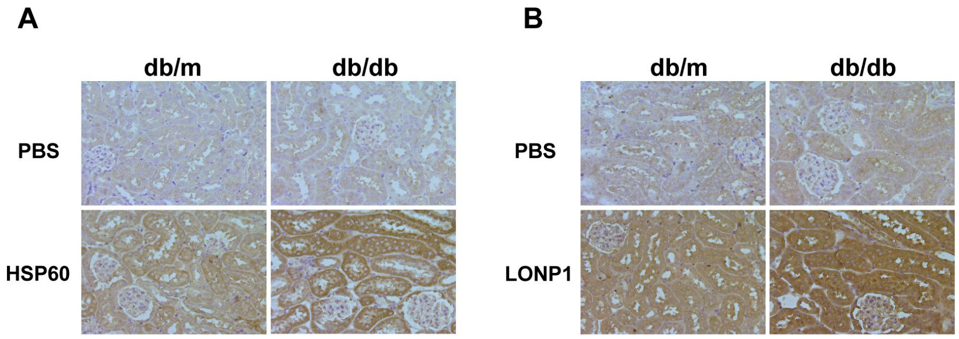

Supplement: Supplementary file 1 — Additional file 1: Figure S1. Transduction efficiency of the Bumpt cellstransfected with LV-ATF5-shRNA. GFP fluorescence imagesand western blotting of ATF5 expressionin Bumpt cells. All data are presented as means ± SD; * p < 0.05 vs LV-NC-shRNA. n = 3. Figure S2. Representative IHC images of negative controland antibody staining. HSP60, LONP1and their negative control of IHC staining in db/m and db/db mice. [file 10020_2023_651_MOESM1_ESM.pdf]
